# Compensation for a Broken Back

**Published:** 1911-12-23

**Authors:** 


### EMPLOYERS' LIABILITY CASES.

### Compensation for a Broken Back.

TOMLINSON *v.* CLIFTON COLLIERY COMPANY.

In this case (Nottingham, October 20) a claim for compensation was put forward by a miner who was said by one medical witness to have walked about for five years with a broken back without suspecting it, until recently, when an *x*-ray photograph revealed it.

In March 1906 Tomlinson was buried under a fall of roof in the mine. Dr. R. Snell went with him to the General Hospital, but neither he nor the house surgeon, Dr. Black, could find a trace of serious injury. Tomlinson walked home, and the next day paralysis set in. He was taken back to the hospital, where he remained a month, and the paralysis disappeared. He was discharged cured, but still suffered pain, and was an out-patient for two years. He had been unable to do any work. "Recently," said Dr. Black, "I examined the man again, took his history, and got evidence that his back was broken. I photographed the spine with the *x*-rays, and it shows unquestionably that the spine is fractured. I hope you will not damage the negative," said the doctor to the judge and counsel, "it is so very interesting from a medical point of view. The man ought not to do any manual work, or the spine may be ricked and he would fall dead."

Dr. Anderson, called on behalf of the colliery company, said he could see nothing in the photograph showing a broken back. He thought the man was suffering from stiffened muscles through being kept still so long. Suitable exercise would do him good. He was in splendid general health. No man with a broken back could have done what Tomlinson did in walking home.

Judge Allen thought the man not wholly incapacitated and awarded him 15s. weekly.

